# Psychosomatics at the threshold: cancer, mood disorders, and the mental pain of death — where precision medicine meets spirituality

**DOI:** 10.3389/fpsyt.2026.1751310

**Published:** 2026-02-10

**Authors:** Mauro Giovanni Carta, Antonio Egidio Nardi

**Affiliations:** 1 Department of Medical Sciences and Public Health, University of Cagliari, Cagliari, Italy; 2 LABPR – Panic and Respiration Laboratory (LABPR), Institute of Psychiatry (IPUB), Federal University of Rio de Janeiro (UFRJ), Rio de Janeiro, Brazil

**Keywords:** cancer, depressive disorders, mental pain, precision medicine, spirituality

## 1 Introduction: cancer and its association with depressive disorder

The comorbidity between chronic illnesses and depression is well known to all psychiatrists working in general hospitals. A large body of research indicates a bidirectional relationship: each condition can exacerbate the other, leading to worse outcomes compared to conditions without comorbidity (1–7). At first glance, the comorbidity between depression and cancer may seem comparable to that seen in other chronic conditions. Yet, a more nuanced reality emerges.

First, this is a phenomenon of considerable scope; however, it is not a distinctive characteristic of cancer (8). According to authoritative large cohort studies, reviews, and meta-analyses, the prevalence of major depressive disorder among people with cancer is estimated within a broad range but is reasonably around 10–15%, varying according to cancer type and the diagnostic tools used (9–11), which is roughly three to four times the rate observed in the general population (12, 13). However, this prevalence is lower than that observed in conditions such as myocardial infarction, in which up to one in three individuals experiences depression (14–16), or diabetes, where approximately one in five individuals is affected (17–20).

Research on cancer stage as a predictor of depression has produced contradictory findings. Some studies report an association between depression and advanced or terminal stages of cancer (21); however, systematic reviews indicate that although advanced or metastatic disease often correlates with a higher burden of depressive symptoms, the effect is modest as a direct determinant and less consistent than psychological or social variables, such as a history of previous depressive episodes or the presence of concurrent stressors (22–24). Observational studies and meta-analyses suggest that depressive symptoms are more frequent during active treatment phases, but findings are heterogeneous and frequently confounded by multiple factors (22, 25).

Based on the current literature, chemotherapy agents and oncological drugs can be broadly classified into high- and moderate-risk categories with respect to depressive disorders. High-risk agents include interferon-α, for which the strongest evidence regarding depression risk derives mainly from non-oncological studies (26, 27), and corticosteroids used in combination therapies, which are associated with an increased risk of mania and mixed states during treatment and depressive episodes following discontinuation (28–30). Agents considered to carry a moderate risk include platinum-based compounds (31), taxanes (32), hormonal therapies for prostate and breast cancer (33, 34), and methotrexate (35).

## 2 Cancer, depressive episodes and quality of life

The apparently lower frequency of the association between cancer and depression compared to what is observed in other chronic diseases does not diminish the importance of comorbidity; rather, the specificity of the depression/cancer relationship becomes evident when the connection between cancer and quality of life is analyzed. A study by our group measured the impact of cancer on quality of life in a consecutive sample of 150 individuals with solid tumors and compared it with the impact of other chronic diseases on quality of life in standardized case-controls studies (21). The research surprisingly revealed that not only is the impact of solid tumors on quality of life no greater than that of other chronic diseases, but also that diseases with a less fatal outcome, such as multiple sclerosis (36) and even fibromyalgia (37), compromise quality of life on average more than solid tumors. The impact was calculated as the difference in the score obtained on the SF-12 scale by individuals with the condition in question, compared to a sample of the same age and sex drawn from the database of an epidemiological study on the well-being of the general Italian population (38). Therefore, all the studies cited by Aviles Gonzales et al. (21) were conducted using a case-control design with controls matched by sex and age to cases, drawn each time from the same database (21). Obviously, some solid tumors have a greater impact than others. In the cited study, the sample comes consecutively from the two regional reference centers in Sardinia, so the proportion of the different tumors is approximately the same as that present in the community.

The relatively low impact (compared to other diseases) of cancer on quality of life might be explained by the fact that, today, suffering from an oncological condition is probably less stigmatizing than it was a few decades ago (39–41), and that treatments and prognosis have improved, including for symptoms such as pain and/or functional disability (42–44). We are increasingly hearing entrepreneurs, politicians, and ordinary citizens speak openly in the media about their experiences after a cancer diagnosis, whereas only a few years ago this was kept secret. Difficulties persist for those who experience ongoing symptoms or live in sociocultural contexts in which stigma is still present. But, paradoxically, despite the relatively high prevalence, one of the greatest challenges concerns depressive disorder. In fact, the same study that highlighted the relatively low impact of cancer on the quality of life of those affected (21) also calculated how the onset of a depressive episode can further reduce the quality of life in people with cancer, and how this compares with other chronic diseases. In other words, we measured the difference in the SF-12 score between those who have cancer or another chronic illness without depression and those who have the same illness but also present depression. It emerges that, in the case of solid tumors, comorbidity with depression reduces quality of life by an average of 10 SF-12 points, which is about three times more than what is observed in other chronic conditions. In cancer, even if the frequency of depression is not very high, when depression is present as a comorbidity, the impact is devastating. The interest of the study by Aviles-Gonzales and colleagues lies in the fact that the comparison between cancer and other chronic diseases was conducted using the same methodology and a shared database derived from a national community survey, allowing the generation of age- and sex-matched control groups for each condition. More broadly, however, the negative impact of depression on cancer outcomes and quality of life is well established in the international literature (45–50).

Living with cancer requires maintaining a delicate balance that hinges, on one hand, on renewed hope for survival made possible by improved treatments, and, on the other, on the acceptance of life’s end as a real possibility, albeit one moderated by therapeutic advances and hope. The onset of a depressive episode can profoundly disrupt this balance, often more severely than in other medical conditions. The combined effects of pain, functional impairment, and stigma, worsened by depression, may lead to a significant loss of hope. In such circumstances, the end of life may appear as inevitable and catastrophic, while the prospect of survival may be perceived instead as a source of anxiety, associated with fears of disability and external dependency.

## 3 Some questions that require answers

The dramatic impact of depressive disorders on quality of life raises significant questions for both research and clinical practice.

1. Is the impact of depressive comorbidity so substantial that it may lead to a worsening of disease progression and even premature death?
2. What mechanisms can be investigated?
3. Is there a bidirectional relationship, namely, can depression increase the risk of cancer?
4. Can depression prevention and early intervention improve outcomes?

We will attempt to propose some answers based on current knowledge. Even in cancer, depression is associated with disease worsening and increased risk of death. A landmark meta-analysis unequivocally revealed, across a large sample of studies, that a diagnosis of depression is linked to a higher mortality rate. This holds true whether depression emerges before or after the cancer diagnosis (51). Subsequent studies have confirmed these findings. In the case of depression–cancer comorbidity, the risk of mortality (compared with individuals with cancer but without depression) is approximately 1.5; however, when mortality not directly related to cancer is also included (for instance, suicide), the risk exceeds 2 (52–55).

Robust evidence found the consequences that depression can exert during the progression of cancer, and how these consequences may serve as mediating factors leading to premature death. For the sake of simplicity, we distinguish between psychosocial and behavioral consequences on the one hand, and biological, somatic consequences on the other; from a psychosomatic perspective, this distinction is artificial, yet we adopt it for clarity.

Among the former, it has been demonstrated that depression leads to poor adherence to treatment (53, 56–58). Depression also reduces energy and motivation, which may result in malnutrition, a sedentary lifestyle, and consequently a general weakening of the organism (55, 59, 60). Individuals with depressive disorders may increase their consumption of alcohol, tobacco, or other risky behaviors (61–63). The loss of social support can further worsen psychological well-being and disease management (64–66).

Among so-called *somatic consequences*, the most significant of which are:

- Alterations of the immune system. Depression may impair immune function, reducing the body’s ability to fight cancer cells (67–69).
- Depression is also associated with elevated levels of inflammatory cytokines, which may promote cancer progression (70–73).
- Depression can disrupt cortisol secretion, negatively affecting the stress response and potentially stimulating tumor growth (74–76).
- Depression can also alter sleep-wake rhythms, resulting in imbalances in several metabolic functions and in the biorhythms of neurotransmitter secretion that influence tumor progression (76, 77).

As can be seen, rather than a clear distinction between psychosocial and somatic consequences, it would be more accurate to speak of two different perspectives from which to observe the same psychosomatic impact.

4, An indefinite border between spirituality and psychosomatics. *Could death wish in depression develop in parallel with the mechanisms responsible for regulating tumor cells?*

The themes of death and depression suggest a psychosomatic perspective that does not exclude a spiritual dimension. It is therefore legitimate to ask whether death wish associated with depression might develop in parallel with the mechanisms responsible for regulating tumor cells. While this question may be relevant from a spiritual standpoint, it also opens the possibility of exploring pathogenic processes. Consequently, one might ask whether, in the absence of an initial tumor, depression, through its physiological effects and the accompanying death wish, could facilitate the onset of cancer. This remains a hypothesis, but it may offer a fruitful direction for both clinical and theoretical investigation.

It’s now widely understood that even people suffering from depression (initially without cancer) are also at increased risk of developing cancer (78–81). The risk is higher in prostate (82–84), breast (85, 86), and lung cancers (87–89). In this framework, we shouldn’t overlook the risk factors produced or amplified by depression, which may act as mediators. This has been well demonstrated in lung cancer, which may represent a model. However, in other cancers, risk factors common to depression and cancer are emerging, particularly breast and prostate cancer, which may represent another model.

## 4 Two different models

The association between depression and lung cancer weakens after adjusting the data for factors such as tobacco use, suggesting that lifestyle (influenced by depression) may play an important mediating role. In this case, it can be stated that the increase in smoking associated with depression is the main cause of the higher incidence of lung cancer among individuals with depression (90). This same model could be useful to explain the depression–cancer association in other organs/anatomical sites. For example, in the Emilia Romagna cohort (91), an elevated risk was also observed for stomach and pancreatic cancer. It would be interesting to investigate whether alcohol consumption plays a similar role in these tumors.

Regarding the second model, an example can be drawn from the fact that mood disorders (92–94), as well as prostate (95, 96) and breast cancer (97, 98), are influenced by light pollution, which disrupts melatonin rhythms, with consequences for circadian hormonal patterns and for the balance between stimulating neurosteroids (estrogens and testosterone) and stabilizing neurosteroids (progestins and their derivatives), in favor of the former (99, 100). Other research findings suggest that a genetic predisposition to mood disorders may be associated with a genetic risk for breast cancer (101). These data do not challenge the hypotheses concerning the impact of light pollution on both conditions (it is perhaps a sensitivity to rhythm dysregulation that is inherited). However, the underlying biological mechanisms deserve further investigation.

## 5 Light pollution, rhythms, and neurotransmitters: does serotonin have a role?

Dorsal raphe neurons, which produce serotonin, display rhythmic electrical activity linked to the sleep–wake cycle (102, 103). Serotonin is a wakefulness-associated neurotransmitter and promotes cell proliferation. This has also been demonstrated in normal, non-cancerous cells (such as epithelial, intestinal, and stam cells) (104, 105). Serotonin may exert a pro-oncogenic role in various types of cancer (106). Some examples include Serotonin stimulates the proliferation of breast cancer cells, which in turn can also produce serotonin themselves (107, 108); Serotonin enhances prostate cancer cell proliferation through the 5-HT1A or 5-HT2B receptors (109). Intestinal serotonin (produced by enterochromaffin cells) has been linked to colon cancer cell proliferation (110). A role for the stress response has also been hypothesized along of a brain-gut axis (111, 112).

Given these premises, it is legitimate to ask whether serotonergic antidepressants may represent a risk factor. Considering all cancer types as a whole, current data suggest that the use of serotonergic medications is not associated with a significant increase in cancer risk, although the available studies are not methodologically robust (113–115). However, when specifically addressing breast cancer, serious concerns arise. A study involving 23,669 breast cancer patients found that SSRI users had higher breast cancer–specific mortality (HR = 1.27; 95% CI 1.16–1.40), which was even higher among long-term SSRI users (HR = 1.54; 95% CI 1.03–2.29). In this case as well, the co-occurrence of depression, although accounted for in the statistical analysis, still leaves room for interpretative ambiguity (116).

## 6 Social support and fear of death

The data presented highlight the importance of psychosocial interventions in preventing the development of depression. A study conducted by our group found that strong psychological and spiritual support, as well as a supportive network of friends, were associated with optimal adherence to cancer treatments and better clinical outcomes (53). Psychosocial interventions and a good social networks are also protective factors against the onset of depressive episodes (117, 118). When antidepressant use was necessary, research on non-cancer depression, showed that psychosocial interventions were associated with lower dosages and shorter treatment durations (119, 120). Therefore, support, even from a single person with whom to share fears and concerns, can play a crucial role, in fact consistent with research on perceived social support in oncology, the perceived availability of at least one emotionally available significant other with whom patients can openly share their fears and concerns functions as an important protective factor against depressive symptoms in people living with cancer (121–123). Even the simple possibility of sharing the fear of death can be a protective factor for both the person suffering from cancer and the caregiver (124, 125).

This concept has also been elegantly expressed through art. In Giovan Battista Caracciolo’s renowned painting *Christ in the Garden of Gethsemane (*https://www.meisterdrucke.ie/fine-art-prints/Giovanni-Battista-Caracciolo/1014752/Christ-on-the-Mount-of-Olives.html*)*, “Battistello” portrays the precise moment in which Christ experiences anguish and fear in the face of imminent death, while simultaneously receiving consolation from an angel. As the Gospel of Luke states: *“Then an angel appeared to him from heaven and strengthened him”* (Luke 22:43). The painting thus conveys two essential elements: on the one hand, the fear of death, restored to its profoundly human dimension; on the other, the presence of someone, the angel, who consoles, shares that fear, and thereby makes it possible to accept it.

To prevent a person with cancer from being aware of the possibility of death, and to deny them dialogue and the sharing of fears, can exacerbate the fear of death as an individual burden that must be concealed rather than shared. Silence intensifies anxiety, obscures awareness of improved treatments, and relegates the possibility of death to a solitary, terrifying event. This suggests that, even in the context of cancer, the possibility of death should not be denied or regarded as inexpressible, provided that the individual wishes to confide. Yet one must ask: does the contemporary world foster loneliness and isolation in the face of death?

## 7 The denial of the awareness of death in contemporary culture

The denial of awareness of death in contemporary culture has attracted the attention of numerous philosophers, sociologists, and intellectuals. According to Philippe Ariès (126), in the modern world death has become a taboo, “invisible,” concealed. In contemporary society, death is suppressed as a social occurrence and delegated to healthcare institutions (126). Michel Foucault argues that modern societies exercise control over life in order to neutralize death as a lived and shared experience, a process he defines as *biopolitical power (2008)*. As a consequence, death is “medicalized” and becomes a technical moment rather than an existential or spiritual one (127).

Zygmunt Bauman adds that in consumer society, death is perceived as an individual failure—something embarrassing that must be hidden (1992). Death no longer carries public meaning; it is reduced to a private, often solitary matter (128). Edgar Morin on “The man and the death” (129) contends that, within contemporary culture, the denial of death generates anxiety, which transforms into an obsession with youth and health, now perceived as synonymous with media-driven beauty.

Prohibiting awareness of the possibility of death in cancer patients, refusing dialogue about it, may ultimately reinforce the fear of death as a personal affront that must be concealed rather than shared.

Death, as a social fact, feared yet regarded as an essential element of life, and from whose fear one may find comfort, lies at the roots of our Mediterranean and Latin cultures. When Georges Duby recounts the life of William “the Marshal,” the quintessential medieval knight, the narrative begins with his death (130). For the medieval man, imbued with a religious and spiritual worldview, life was fundamentally a preparation for death.

Similarly, in Islamic ethical-religious literature—particularly within Sufi thought—earthly life is understood as a preparation (*fitna*) for the hereafter (*akhira*) (131).

Even atheist or agnostic philosophers such as Sartre (132)Cioran (133), and Camus (134) developed the theme of death as a search for the meaning of life, or as a form of existential contemplation. “To die proudly when it is no longer possible to live proudly” reflects an exacerbation of individualism, yet it also implies a need for social testimony.

The COVID-19 pandemic demonstrated how, within Mediterranean societies, the prohibition of funeral rites was associated with an increased risk of depression among relatives of the deceased. demonstrating in this way how the sociality of death can be an essential moment in the metabolization of mourning (135).

Despite the media bombardment that denies death as an individual value and a social event (as well as the social importance of death and life), this vision has not yet fully taken root in our cultures. The experience of cancer can be lived with reasonable hope thanks to the successes of science, but it also requires reflection that can give meaning to life, not only because the fear of possible death is modulated by hope, but also because of the social significance of the disease, consistent with our cultural roots. Those who have already lived this experience (healthcare workers or caregivers) can more easily be a point of reference for those who are going through.

## 8 A summary with practical suggestions from this complex perspective

Prevention of depressive episodes is essential: attention should be paid to signs of stress and to dysregulation of social rhythms; sometimes the alteration of the sleep–wake cycle alone may serve as an early warning signal. Providing accurate information to patients and their caregivers is equally important. A crucial aspect is also the organization of services and the provision of *comprehensive* care—too often individuals are left on their own to navigate clinical examinations, specialist imaging, and other procedures.

Emotional support plays a significant role. Support groups, including self-help groups, may be valuable, particularly (though not exclusively) for individuals who feel isolated or lack a social network. It may be beneficial to have someone with whom, due to cultural or spiritual proximity, it is easier to discuss these issues.

If a depressive episode is diagnosed, it is important to keep in mind that several forms of psychotherapy are effective. Pharmacotherapy should not be avoided when necessary; however, the most appropriate medications should be carefully evaluated, and, whenever possible, treatment duration should not be unnecessarily prolonged (as previously discussed with regard to serotonin). For this reason, psychotherapy or other interventions that may help shorten the duration of pharmacological treatment are essential. It must be borne in mind that depression is a multifactorial disorder and therefore requires diversified therapeutic approaches, not limited to psychopharmacological treatment alone; moreover, effective non-pharmacological interventions have also been tested and shown to be beneficial in oncology settings (136–139).

This reading has considered (the treatment and prevention of) the disease as a social event, both in terms of the importance of the specific cultural context as a place of care and a tool for treatment, and as a social mediator for prevention (105-142). This does not deny the relevance of disease in a globalized context, nor does it contrast this with an awareness of the role of technology and,science but rather, it integrates technology into cultural contexts.
